# Integrated transcriptomic and variant analysis reveals molecular mechanisms of pyrethroid resistance in a genetically homogenized Cas9 strain of *Aedes aegypti*

**DOI:** 10.1007/s00204-026-04326-x

**Published:** 2026-03-16

**Authors:** Dylan Brown, Reese Houck, Nannan Liu

**Affiliations:** Department of Entomology and Plant Pathology, School of Agriculture, Auburn University, Auburn, AL 36849 USA

**Keywords:** *Aedes aegypti*, Pyrethroid resistance, Transcriptomics, SNP analysis, Genetically homogenized Cas9 strain, Resistance mechanisms

## Abstract

Insecticide resistance, a central problem in insecticide toxicology, in *Aedes aegypti* continues to undermine vector control efforts worldwide. Although transcriptomic studies have identified numerous resistance-associated genes, differences in genetic background between resistant and susceptible strains often obscure which changes are directly linked to resistance. To reduce background variability, we generated a genetically homogenized pyrethroid-resistant Cas9 strain (PRCas9) by repeatedly backcrossing a susceptible Cas9 strain (exu-Cas9 line) with a highly permethrin-resistant Puerto Rico (PR) strain. PRCas9 retained extreme resistance comparable to the parental PR strain (> 18,000-fold resistance ratio) and was further analyzed alongside the PR and Cas9 strains using RNA-seq. Using this controlled genetic background, transcriptomic profiling identified > 70 differentially expressed genes between resistant and susceptible mosquitoes, revealing reproducible resistance-associated detoxification in insecticide toxicology signatures dominated by cytochrome P450s and carboxylesterases. qRT-PCR validated elevated expression of key detoxification candidates. Variant and promoter sequencing further revealed unique SNPs in detoxification genes, including *CYP9J23*, *CYP9J28*, and venom carboxylesterase-6, as well as distinct variants in NOX4-art, a NADPH oxidase involved in reactive oxygen species (ROS) production. Gene ontology analysis supported enrichment of detoxification-related molecular functions. Increased NOX4-art expression, together with these regulatory variants, suggests a potential role for ROS-linked regulatory pathways in coordinating detoxification gene activation. This study demonstrates the value of a genetically homogenized resistant Cas9 background for dissecting resistance mechanisms while identifying candidate genes and regulatory elements contributing to pyrethroid resistance. PRCas9 provides a powerful platform for future CRISPR-based functional analyses in insect toxicology aimed at improving vector control strategies.

**Supplementary Information:**

The online version contains supplementary material available at 10.1007/s00204-026-04326-x.

## Introduction

The yellow fever mosquito, *Aedes aegypti*, is a major vector of chikungunya, yellow fever, dengue, and Zika viruses, which together infect tens of millions of people each year (Nwaiwu et al. 2021; Lenharo 2023). Native to Africa, *Ae. aegypti* has spread to nearly all continents through human-mediated transport (Jansen and Beebe 2010). Although *Ae. aegypti* typically thrives in urban, subtropical regions, recent climate and environmental shifts have enabled its expansion into temperate areas (Iwamura et al. 2020). As a result, the geographic risk of arbovirus transmission continues to grow, underscoring the need for reliable vector control strategies within the framework of insect toxicology.

Chemical control remains central to mosquito management programs, with pyrethroids being among the most widely used insecticides in insect toxicology due to their rapid neurotoxicity and low mammalian risk (Smith et al. 2016). Pyrethroids act by binding to voltage-gated sodium channels (VGSCs), prolonging channel opening and disrupting neuronal signaling, which leads to paralysis and death of the insect (Du et al. 2013; Pinch et al. 2020). However, intensive use of these compounds has been selected for high levels of resistance in *Ae. aegypti* populations worldwide (Reid et al. 2014; Dusfour et al. 2015; Goindin et al. 2017; Ishak et al. 2017; Lien et al. 2019; Rault et al. 2019; Derilus et al. 2023; Brown et al. 2025). Resistance in insect toxicology is primarily driven by three mechanisms: mutations in VGSC gene that reduce pyrethroid binding (kdr) (Liu 2015; Ponce et al. 2016; Barrera-Illanes et al. 2024), elevated metabolic detoxification by cytochrome P450s, GSTs, UGTs, and esterases (Stevenson et al. 2011; Ali et al. 2020; Li et al. 2020), and changes in cuticular structure that limit insecticide penetration (Vannini et al. 2014; Balabanidou et al. 2016).

Transcriptomic approaches have become essential in toxicogenomic studies for identifying genes involved in insecticide resistance. By comparing resistant and susceptible populations, researchers can identify differentially expressed genes, including cytochrome P450s, GSTs, carboxylesterases, UGTs, and ABC transporters (Lien et al. 2019; Ettinger et al. 2022). Transcriptomic data can also reveal single nucleotide polymorphisms (SNPs) that alter enzyme activity or gene regulation (Xu et al. 2018; Li et al. 2012). For example, a single SNP in the *GSTe2* gene confers strong DDT resistance in *Anopheles funestus* (Riveron et al. 2014). Together, these insights highlight the value of transcriptomics in identifying both expression-level changes and genetic variants that contribute to resistance.

Our previous studies using synergist bioassays and *vgsc* sequencing showed that both kdr mutations and metabolic detoxification, particularly cytochrome P450s and carboxylesterases, contribute to permethrin resistance in *Ae. aegypti* (Wang et al. 2023c). However, the specific metabolic genes involved in permethrin detoxification have remained unclear. To address this limitation, we performed transcriptomic analyses on three populations: a pyrethroid-susceptible Cas9-modified Liverpool strain, the pyrethroid-resistant Puerto Rico (PR) strain, and a novel PRCas9 strain generated through repeated backcross of Cas9 onto the PR background. PRCas9 retains the high permethrin resistance of the PR strain while providing a genetically tractable Cas9 background suitable for functional genetic studies. Transcriptomic sequencing revealed multiple overexpressed detoxification genes and identified unique SNPs within coding and promoter regions of several candidates. These findings provide new insights into the molecular basis of pyrethroid resistance in *Ae. aegypti* and highlights targets for future functional validation and resistance management strategies.

## Material and methods

### Mosquito populations

Four *Ae. aegypti* populations were used in this study. The susceptible Orlando strain (ORL) has been maintained at the Center for Medical, Agricultural, and Veterinary Entomology (CMAVE), USDA-ARS in Gainesville, FL, since 1952 (Allan 2011). The Cas9 strain used in this study was the exu-Cas9 line described by Li et al. (2017), originally derived from the Liverpool strain, which expresses Cas9 under the *exuperantia* promoter and carries a dominant Opie2-DsRed fluorescent marker for transgene identification. This line was kindly provided by Dr. Omar Akbari (University of California, Riverside). The Puerto Rico (PR) strain was collected in urban San Juan, Puerto Rico, in 2008 (Reid et al. 2014; Estep et al. 2017). ORL and PR were kindly provided by Dr. Jimmy Becnel (CMAVE). To increase permethrin resistance, the PR strain underwent 22 generations of permethrin selection (2–3 times per year since 2013). This strain carries two well-characterized kdr mutations, V1016I and F1534C, in the voltage-gated sodium channel gene, both in the homozygous state, and both known as contributors to pyrethroid resistance in *Ae. aegypti*.

**Fig. 1:**
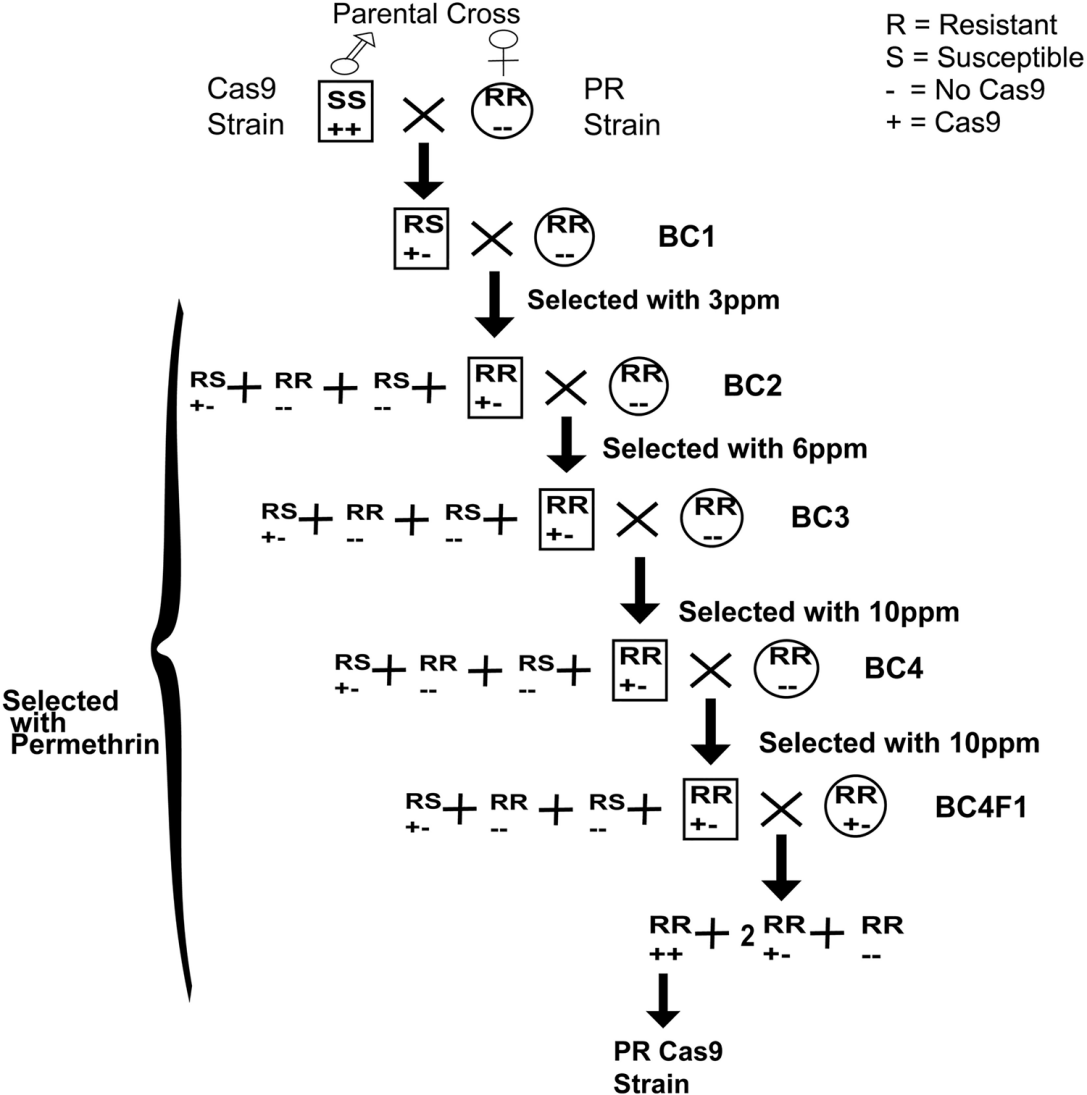
Backcross scheme for generating the pyrethroid-resistant PRCas9 strain of *Aedes aegypti*. Two reciprocal crosses were performed: Cas9 males × PR females and PR males × Cas9 females. Offspring from each generation were subjected to increasing concentrations of permethrin for four successive generations. Survivors were screened for red fluorescence to confirm inheritance of the Cas9 gene

The PRCas9 strain was developed by repeatedly backcrossing Cas9-positive individuals to the PR strain (Fig. 1). Reciprocal backcrosses (PR male × Cas9 female, PR female × Cas9 male) were performed to ensure stable incorporation of the Cas9 transgene. Although the PR strain has undergone long-term laboratory selection, it remains a genetically consistent model for reproducible mechanistic studies. The aim of generating PRCas9 was to create a highly resistant, genetically homogenized Cas9 background suitable for controlled functional and transcriptomic analyses, rather than to represent contemporary field populations. All mosquito populations were reared at 25 ± 2 °C under a 12:12 h light:dark photoperiod.

### Larval bioassays

Larval bioassays followed the protocol of Wang et al. (2023b). Minor modifications were made to accommodate experimental design and sample size. Permethrin [3-phenoxybenzyl (1RS,3RS;1RS,3SR)-3-(2,2-dichlorovinyl)-2,2-dimethyl-cyclopropanecarboxylate, 94.34%] was provided by FMC Corporation (Princeton, NJ). Briefly, Bioassays were conducted in 20 mL disposable scintillation vials by first adding 5 mL of tap water. Ten similar sized late third-early fourth instar larvae were transferred to each vial, followed by an additional 5 mL of tap water to reach a total volume to 10 mL. Less than 100 µL of insecticide solution of permethrin was added to each vial to obtain the desired concentrations. Control vials received an equivalent volume of acetone.

Each bioassay was replicated at least three times on separate days under the same environmental conditions used for mosquito rearing. Although pyrethroids are primarily applied against adult mosquitoes, larvae were selected for both bioassays and transcriptomic analyses because they are developmentally uniform, easy to handle, and suitable for high-quality RNA extraction. Larvae have also been widely used to characterize constitutive expression of detoxification genes associated with resistance (Saavedra-Rodriguez et al. 2012; David et al. 2014; Morgan et al. 2022; Helvecio et al. 2025).

Bioassay data was analyzed by probit analysis using Polo-PC software. Resistance ratios (RRs) were calculated by dividing the LC₅₀ of each resistant strain by the LC₅₀ of the susceptible ORL strain. ORL served as the reference baseline to remain consistent with prior toxicology studies involving the PR strain and to avoid any influence of the Cas9 transgene on resistance ratio calculations. Statistical comparisons of LC₅₀ values were based on non-overlapping 95% confidence intervals (Wen and Scott 1997). Resistance ratios ˃10 were indicative of high resistance, while values < 10 indicated relative susceptibility (Valles et al. 1997; Wen and Scott 1997; Liu et al. 2004).

### Transcriptomic sequencing

To identify genes associated with pyrethroid resistance in the PR and PRCas9 populations, we performed RNA sequencing (RNA-seq) on late-third/early-fourth instar larvae. For each strain (Cas9, PR, and PRCas9), sixty larvae were flash-frozen per replicate, and three independent biological replicates were collected. Each replicate consisted of larvae reared in separate containers on different days from distinct egg batches. Samples were submitted to Novogene for RNA extraction and sequencing using the NovaSeq PE150 platform, generating 150 bp paired-end reads. Raw reads were trimmed using Trimmomatic v0.39 with the following parameters: HEADCROP:10 LEADING:30 TRAILING:30 SLIDINGWINDOW:6:30 MINLEN:36, to remove low-quality reads and adapter sequences (Bolger et al. 2014). Read quality before and after trimming was assessed with FASTQC. Clean reads were aligned to the *Ae. aegypti* reference genome (AaegL5_3) using HISAT2 (Kim et al. 2019). Read quantification was performed using StringTie (Pertea et al. 2015), and differential expression analysis was performed using the DESeq package in R (Love et al. 2014). Genes were considered differentially expressed if they met two criteria: log₂ fold-change (log₂FC) ˃1, and an adjusted *p*-value less than 0.05. *P*-values were corrected using the Benjamini–Hochberg false discovery rate (FDR) method. Volcano plots of differentially expressed genes were generated using ggplot2 in R version 4.2.3 (R Core Team and Team 2024).

For variant analysis, aligned reads were processed using Picard tools (http://broadinstitute.github.io/picard) to mark duplicates (MarkDuplicates), assign read groups (AddOrReplaceReadGroups), split reads containing N’s (SplitNCigarReads). Variant calling was performed with GATK v4.0 Haplotype Caller, followed by VariantFiltration using the parameters QD > 2.0, MQ > 40.0, FS < 60.0, SOR < 3.0, MQRankSum > –12.5, and ReadPosRank > –8.0 (Van der Auwera and O’Connor 2020). SNP effects were annotated with snpEff v4.3 (Cingolani et al. 2012). Variants were retained only if they had a minimum read depth ≥ 3 and an allele frequency ≥ 90%.

### Amplification and sequence analysis of variants and CYP450 promoter regions

Genomic DNA was extracted from 15 late third-early fourth instar larvae from each of populations (Cas9, PR, and PRCas9) using the DNeasy Blood and Tissue Kit (QIAGEN, Venlo, Netherlands). Five genes containing a total of 20 SNPs were selected for variant validation. Primer pairs were designed to amplify exonic regions of each gene containing the target SNPs (Supplementary Table S1). To identify additional SNPs and insertions/deletions (INDELs) in overexpressed P450 genes, primers were also designed to amplify approximately 1,000 base pairs upstream of the start codon for each gene. PCR products were purified using the QIAquick PCR Purification Kit (QIAGEN, Venlo, Netherlands) and submitted for Sanger sequencing (GENEWIZ, South Plainfield, NJ). Primer sequences used for promoter region amplification are listed in Supplementary Table S1.

### Quantitative real-time polymerase chain reaction (qRT-PCR)

Six upregulated cytochrome P450 genes and four carboxylesterase genes were selected for qRT-PCR validation. Ten late-third/early-fourth instar larvae were randomly collected from each population and flash-frozen on dry ice to generate one biological replicate. Total RNA was extracted using the RNeasy Kit (QIAGEN, Venlo, Netherlands), and genomic DNA was removed using the TURBO DNA-free Kit (Ambion) following the manufacturer’s instructions. First-strand cDNA was synthesized from 5 μg of total RNA using the SuperScript IV First-Strand Synthesis System (Invitrogen, Carlsbad, CA) with random hexamer primers.

qRT-PCR was performed using the SYBR Green PCR Master Mix (Roche) on a CFX96 Real-Time PCR Detection System (Bio-Rad, Hercules, CA, USA). Each 10 µL reaction contained 1 × SYBR Green master mix, 1 µL of cDNA (2.5 ng), and gene-specific primers (Supplementary Table S1) at a final concentration of 0 µM. The 60S ribosomal RNA gene (*AAEL000987*) was used as the internal control because its expression remained stable across resistant and susceptible populations. The thermal cycling conditions were 95 °C for 120 s, followed by 40 cycles of 95 °C for 5 s and 60 °C for 30 s. Three independent biological replicates were conducted for each population.

## Results

### Generation of permethrin resistant Cas9 strain of *Ae. aegypti*

To generate a highly pyrethroid-resistant *Ae. aegypti* Cas9 strain with a uniform genetic background, a susceptible Cas9 strain was repeatedly backcrossed with the pyrethroid-resistant PR strain (Fig. 1). The parental PR strain exhibited approximately 19,000-fold higher permethrin resistance than the susceptible ORL. Two reciprocal crosses were performed to assess potential sex-linked inheritance of resistance: PR females were crossed with Cas9 males, and Cas9 females were crossed with PR males. After successive rounds of backcrossing under increasing permethrin selection pressure, the resulting progeny strains displayed resistance ratios ranging from ~ 23,000- to 30,000-fold relative to ORL (Table 1). Overlapping LC₅₀ confidence intervals indicated no evidence of sex-linked inheritance of pyrethroid resistance.

**Table 1:** Toxicity of permethrin to different mosquito strains of *Ae. aegypti*

Strain	df	n	X2	LC50(CI)	LC90 (CI)	Slope (SE)	RR*
ORL	2	119	0.13	0.015 (0.012–0.019)	0.032 (0.025–0.052)	3.95 ± 0.71	1
Cas9	2	120	0	0.028 (0.026–0.031)	0.036 (0.032–0.045)	12.35 ± 2.84	1.9
PR	1	80	0.012	280 (220–440)	680 (440–2300)	3.35 ± 0.85	19,000
PR Female x Cas9 Male	1	86	0.38	450 (280–2,100)	1800 (720–76,000)	2.10 ± 0.66	30,000
PR Male x Cas9 Female	1	80	0.03	340 (250–740)	960 (530–7500)	2.85 ± 0.83	23,000

^*^RR: Resistance Ratio (LC_50_ of resistant stain divided by LC_50_ of susceptible strain ORL)

Although the final resistance levels were comparable between the two lines derived from the reciprocal backcrosses, we observed a notable difference in the inheritance rate of the Cas9 gene. Cas9 is marked with a *dsRed* fluorescence reporter, enabling efficient screening of pupae. Following each round of backcrossing and selection, fluorescent pupae were counted to determine inheritance frequency. The PR females x Cas9 males yielded offspring with an average Cas9 inheritance rate of 78%, whereas the reciprocal cross (PR males × Cas9 females) yielded a significantly lower rate of 44% (Table 2). This difference was statistically significant (χ^2^ = 62.87; df = 1; P < 0.001), indicating a sex-associated transmission bias of the Cas9 transgene. While the exu-Cas9 insertion site on chromosome 3 has been reported previously (Li et al. 2017), the absence of insertion-site mapping in this study means that additional insertion(s) or linkage to sex-determination loci (e.g., *nix*) cannot be excluded and may contribute to the observed inheritance pattern. Future genomic mapping could help further resolve the genetic basis of this transmission bias.

**Table 2:** Inheritance rate of the Cas9 gene after repeated backcross and permethrin selection

Generation	Genetic Cross	Cas9 Inheritance Rate*
BC1	PR Female x BC1 Male	74% (194/261)
	PR Male x BC1 Female	47% (192/410)
BC2	PR Female x BC2 Male	80% (154/204)
	PR Male x BC2 Female	42% (198/472)
BC3	PR Female x BC3 Male	77% (205/266)
	PR Male x BC3 Female	45% (98/218)
BC4	PR Female x BC4 Male	79.3% (169/213)
	PR Male x BC4 Female	42% (201/484)

^*^ Calculated as the number of fluorescent individuals divided by the total number of mosquitoes screened

### Transcriptomic sequencing

Illumina sequencing generated approximately 60–80 million paired-end reads per stains (Table 3). After trimming, ~ 88% of high-quality paired-end reads were retained for downstream analyses. The average pre-trim read length was 150 bp, and post-trim read lengths ranged from 36 to 140 bp. Quality score exceeded Q36 both before and after trimming. Overall, 92% of reads mapped successfully to the *Ae. aegypti* reference genome (AaegL5_3).

**Table 3:** *Ae. aegypti* RNAseq data before and after cleaning

Sample ID	# of reads before cleaning	# of reads after cleaning	Average quality score preclean	Average quality score postclean	Sequence length preclean	Sequence length postclean
Cas9	68,808,116	60,175,430	36	36	150	36–140
PR	72,966,529	64,492,296	36	36	150	36–140
PRCas9	78,763,690	69,971,761	36	36	150	36–140

**Fig. 2:**
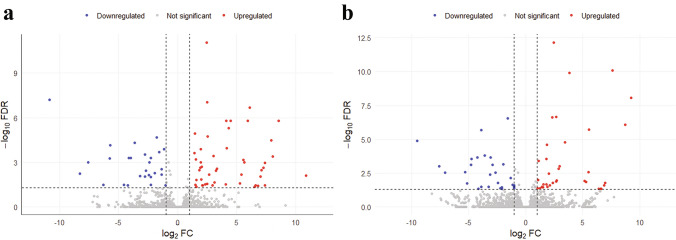
Volcano plot showing differentially expressed genes (DEGs) between resistant susceptible Cas9 population and a) PR and b) PRCas9. Each dot represents a gene. The x-axis shows the log₂ fold-change in gene expression (i.e., how much a gene’s expression increased or decreased), and the y-axis shows the statistical significance of that change (–log₁₀ of the adjusted *p*-value). Genes with at least a two-fold change in expression (|log₂FC|> 1) and statistically significant differences (*adjusted p* < 0.05) are considered differentially expressed. Red dots represent significantly upregulated genes in resistant populations, blue dots represent significantly downregulated genes, and gray dots indicate genes with no significant change

Relative to the susceptible Cas9 strain, 83 and 71 genes were differentially expressed in the PR and PRCas9 strains, respectively, with 51 and 40 upregulated and 32 and 31 downregulated genes (Fig. 2). Genes were classified as differentially expressed if they met two criteria: (1) log₂ fold change (log₂FC) > 1 or < –1, corresponding to at least a two-fold change in expression, and (2) an adjusted *p*-value < 0.05, after Benjamini–Hochberg correction. These thresholds ensured that only genes with both biologically meaningful and statistically significant expression differences were included in downstream analyses and in the volcano plot (Fig. 2). Among the differentially expressed genes, seven cytochrome P450 (*CYP450*) genes were shared between PR and PRCas9 strains, with 3 being overexpressed while 4 were under expressed in the resistant populations. One esterase gene was upregulated in both resistant strains, and two additional esterase genes were only upregulated in the PR and not PRCas9 strain.

**Fig. 3:**
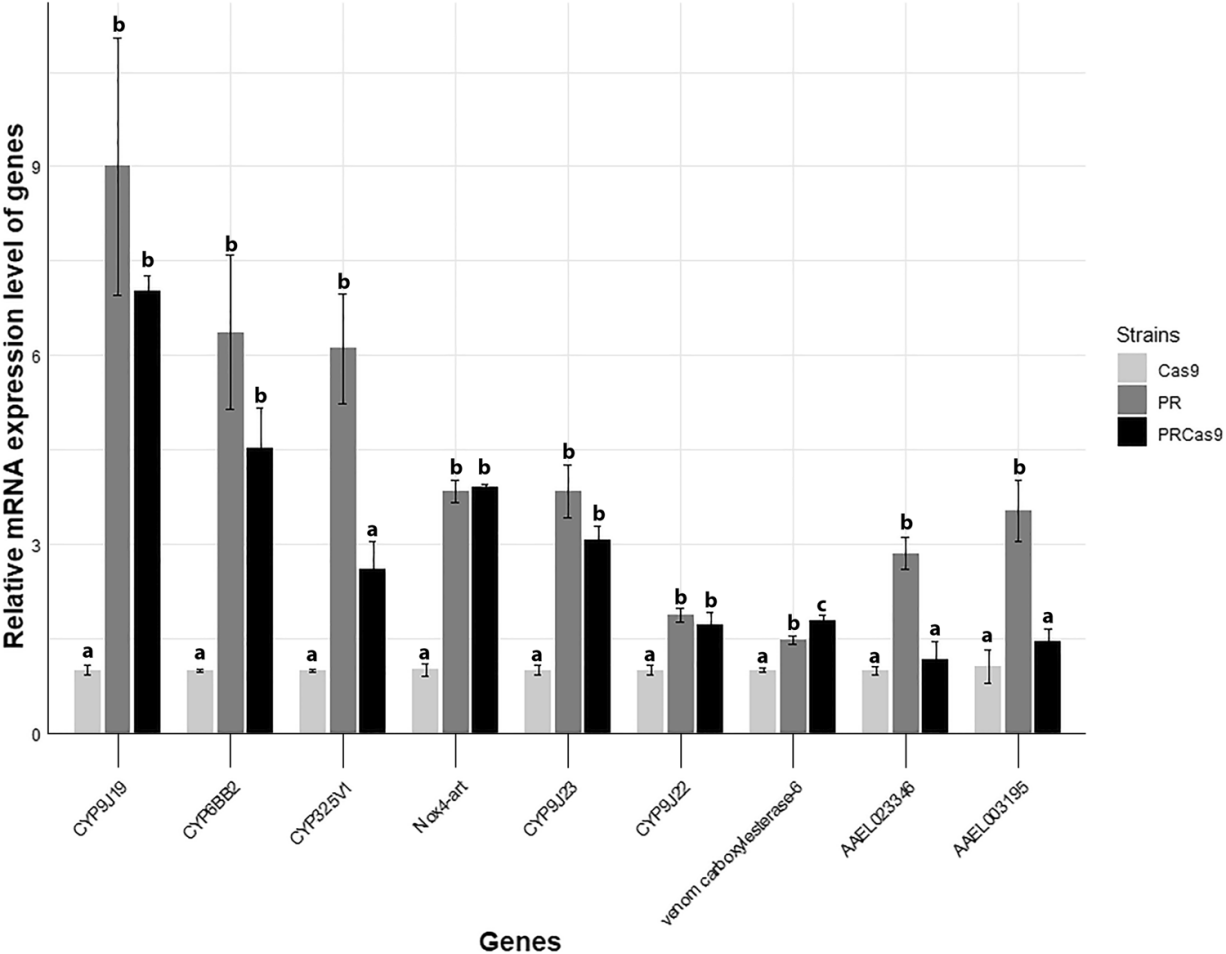
qRT-PCR expression analysis of ten candidate genes: *CYP9J19, CYP6BB2, CYP325v1, NOX4-art, CYP9J23, CYP9J22, venom carboxylesterase-6, AAEL023346, AAEL003195*. The Y-axis indicates relative expression levels (fold change) in resistant versus susceptible strains. Data are presented as mean ± standard error (S.E.). All genes were significantly overexpressed in resistant populations (P ≤ 0.05)

qRT-PCR was used to validate selected transcriptomic findings, including genes significantly upregulated in both resistant strains, (*CYP6BB2* [AAEL014893], *CYP9J23* [AAEL014615], *CYP9J19* [AAEL028635], *venom carboxylesterase-6* [AAEL008757]), genes upregulated in only one resistant strain (*CYP325v1* [AAEL017136], *CYP9J22* [AAEL014619], *fatty acyl-CoA hydrolase precursor, medium chain-like* [AAEL023346], and *carboxylic ester hydrolase* [AAEL003195]). *NOX4-art* (AAEL022189) was also validated using qRT-PCR although it had log₂FC > 1 but an adjusted p-value > 0.05. qRT-PCR confirmed significant overexpression of *CYP6BB2*, *CYP9J19*, *CYP9J22*, *CYP9J23*, and *venom carboxylesterase-6* in both PR and PRCas9 strains compared to the susceptible Cas9 strain (Fig. 3). Consistent with the transcriptomic data, *CYP325v1*, fatty acyl-CoA hydrolase precursor, medium chain-like, and Carboxylic ester hydrolase were significantly upregulated only in the PR strain (Fig. 3). qRT-PCR showed significant upregulation of *Nox4-art* (AAEL022189) in both PR and PRCas9, whereas transcriptomic data indicated a similar magnitude of change (log2FC > 1) but with adjusted p-value > 0.05. In addition, although *CYP9J22* (AAEL014619) showed significant upregulation only in PR in the RNA-seq dataset, qRT-PCR confirmed its overexpression in both resistant strains, suggesting higher sensitivity of qRT-PCR for detecting moderate expression differences (Table 4).

**Table 4:** Summary of potential metabolic detoxification genes that had a log2-fold change of 1 or greater in at least one pyrethroid resistant strain

Gene	VectorBase accession No	PR	PRCas9
*NOX4-art*	AAEL022189	1.3*	1.3*
*CYP6BB2*	AAEL014893	2.0	1.3
*CYP9J19*	AAEL028635	2.0	1.8
*CYP9J22*	AAEL014619	0.63	1.1
*CYP9J23*	AAEL014615	2.5	2.3
*CYP325V1*	AAEL017136	6.8	7.2*
*Venom carboxylesterase-6*	AAEL008757	1.4	1.1
*Fatty acyl-CoA hydrolase precursor, medium chain-like*	AAEL023346	1.5	1.3*
*Carboxylic ester hydrolase*	AAEL003195	1.6	1.4*

^*^ Log2-Fold change was greater than 1 but the adjusted p-value was greater than 0.05

### Variant analysis

**Fig. 4:**
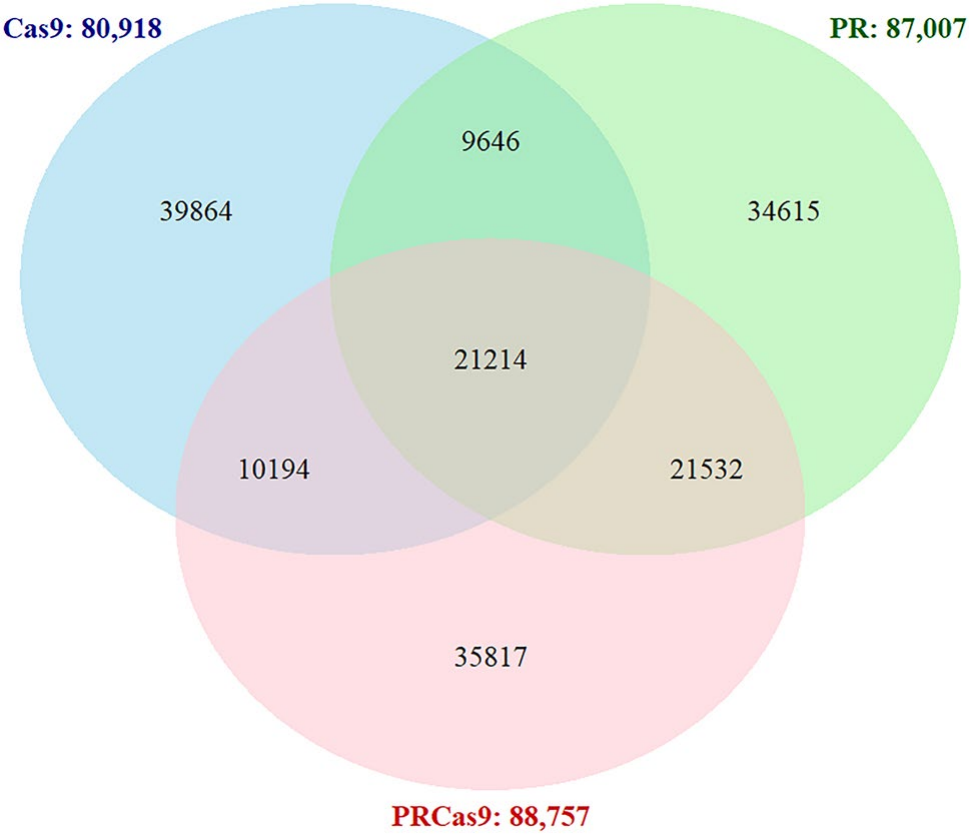
SNP analysis in resistant (PR and PRCas9) and susceptible (ORL and Cas9) *Aedes aegypti* strains. (A) Venn diagram showing the overlap and unique SNPs in each population relative to the reference genome (*AaegL5_3*)

A total of 80,918, 87,007 and 88,757 SNPs (allele frequency of ≥ 90%; read depth ≥ 3) were identified from the transcriptomic data in the Cas9, PR, and PRCas9 strains, respectively (Fig. 4), relative to the reference Liverpool reference genome (AaegL5_3). Approximately 24% SNPs were shared between the PR and PRCas9 populations, while ~ 11% were shared with the Cas9 strain. Among the SNPs unique to PR and PRCas9 populations, several were located in differentially expressed metabolic detoxification genes, including P450s (*Nox4-Art, CYP9J19, CYP9J22, and CYP9J23*) and esterases (*venom carboxylesterase-6 and carboxylic ester hydrolase)*.

To validate transcriptome-derived SNPs, Sanger sequencing was performed on five metabolic detoxification genes containing 20 predicted SNPs (*CYP9J23, CYP9J28, venom carboxylesterase-6, glutactin, and ABC transporter*). Eighteen of these SNPs showed single, clear peaks in both PR and PRCas9, confirming that these variants are fixed in the resistant populations (Table 5). In contrast, both missense mutations, A to G and T to A, in *CYP9J28* displayed mixed peaks, indicating that these mutations remain unfixed in the resistant strains. In the Cas9 strain, 14 of the 20 SNPs were heterozygous, consistent with the absence of selection pressure. The remaining 6 SNPs were completely absent in the Cas9 strain, indicating that they are unique to the PR and PRCas9 populations.

**Table 5:** Synonymous and nonsynonymous variants with a minimum read depth ≥ 3 and an allele frequency ≥ 90% further validated through Sanger sequencing

Gene	Transcript ID	Mutation	Cas9	PRCas9	PR
CYP9J23	AAEL014615	Nonsynonymous mutation at position 368564364 (G- > A)			
CYP9J23	AAEL014615	Nonsynonymous mutation at position 368564865 (A- > G)			`
CYP9J23	AAEL014615	Nonsynonymous mutation at position 368565212 (C- > G)			
CYP9J23	AAEL014615	Synonymous mutation at position 368564932 (T- > G)			
CYP9J23	AAEL014615	Synonymous mutation 368564656 (C- > T)			
CYP9J23	AAEL014615	Synonymous mutation at position 368564785 (T- > C)			
CYP9J28	AAEL014617	Nonsynonymous mutation at position 368628215 (A- > G)			
CYP9J28	AAEL014617	Nonsynonymous mutation at position 368627876 (T- > A)			
Venom carboxylesterase-6	AAEL008757	Nonsynonymous mutation at position 326025231 (G- > A)			
Venom carboxylesterase-6	AAEL008757	Nonsynonymous mutation at position 326024706 (G- > A)			
Venom carboxylesterase-6	AAEL008757	Nonsynonymous mutation at position 326024616 (G- > A)			
Venom carboxylesterase-6	AAEL008757	Nonsynonymous mutation at position 326024387 (C- > G)			
Venom carboxylesterase-6	AAEL008757	Synonymous mutation at position 326024371 (C- > G)			
Carboxylic ester hydrolase	AAEL000904	Nonsynonymous mutation at position 332973542 (G- > A)			
Carboxylic ester hydrolase	AAEL000904	Nonsynonymous mutation at position 332973538 (T- > C)			
Carboxylic ester hydrolase	AAEL000904	Nonsynonymous mutation at position 332973534 (G- > C)			
Carboxylic ester hydrolase	AAEL000904	Nonsynonymous mutation at position 332972349 (A- > T)			
Carboxylic ester hydrolase	AAEL000904	Nonsynonymous mutation at position 332971750 (A- > G)			
ABC transporter	AAEL008624	Nonsynonymous mutation at position 45564452 (T- > C)			
ABC transporter	AAEL008624	Nonsynonymous mutation at position 455649295 (T- > C)			

**Fig. 5:**
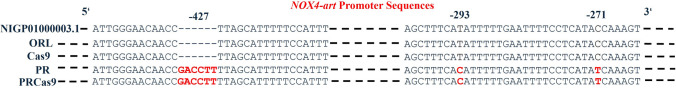
Sequence alignment of the promoter region of *NOX4-art* (*AAEL022189*) among four *Aedes aegypti* strains. A ~ 1000 bp region upstream of the start codon was analyzed in ORL, Cas9, PR, and PRCas9 strains. Alignment was performed using the CLUSTAL algorithm. Unique SNPs and a 6-bp deletion found in resistant strains (PR and PRCas9) are highlighted in red

To investigate potential regulatory mechanisms underlying the overexpression of P450 genes in resistant strains, promoter regions (~ 1 kb upstream of start codon) of five overexpressed P450 genes (*CYP325v1, CYP9J23, CYP6BB2, CYP9J19, and CYP9J22*) and one NOX4-art (*AAEL022189)* were sequenced. Among these, only the promoter regions for CYP9J19 and *NOX4-Art* contained variants unique to PR and PRCas9 that were not found in the susceptible Cas9 strain. The *CYP9J19* promoter carried 15 SNPs, while the *NOX4-art* promoter contained eight SNPs and a six-base-pair deletion relative to the susceptible ORL, Cas9, and reference genome (Fig. 5). *NOX4-Art* encodes an NADPH oxidase responsible for generating hydrogen peroxide, a reactive oxygen species (ROS), in *Ae. aegypti* (Gandara et al. 2017). The extensive promoter variation observed in *NOX4-Art* may contribute to altered transcriptional regulation and elevated expression in resistant populations.

### GO analysis

**Fig. 6:**
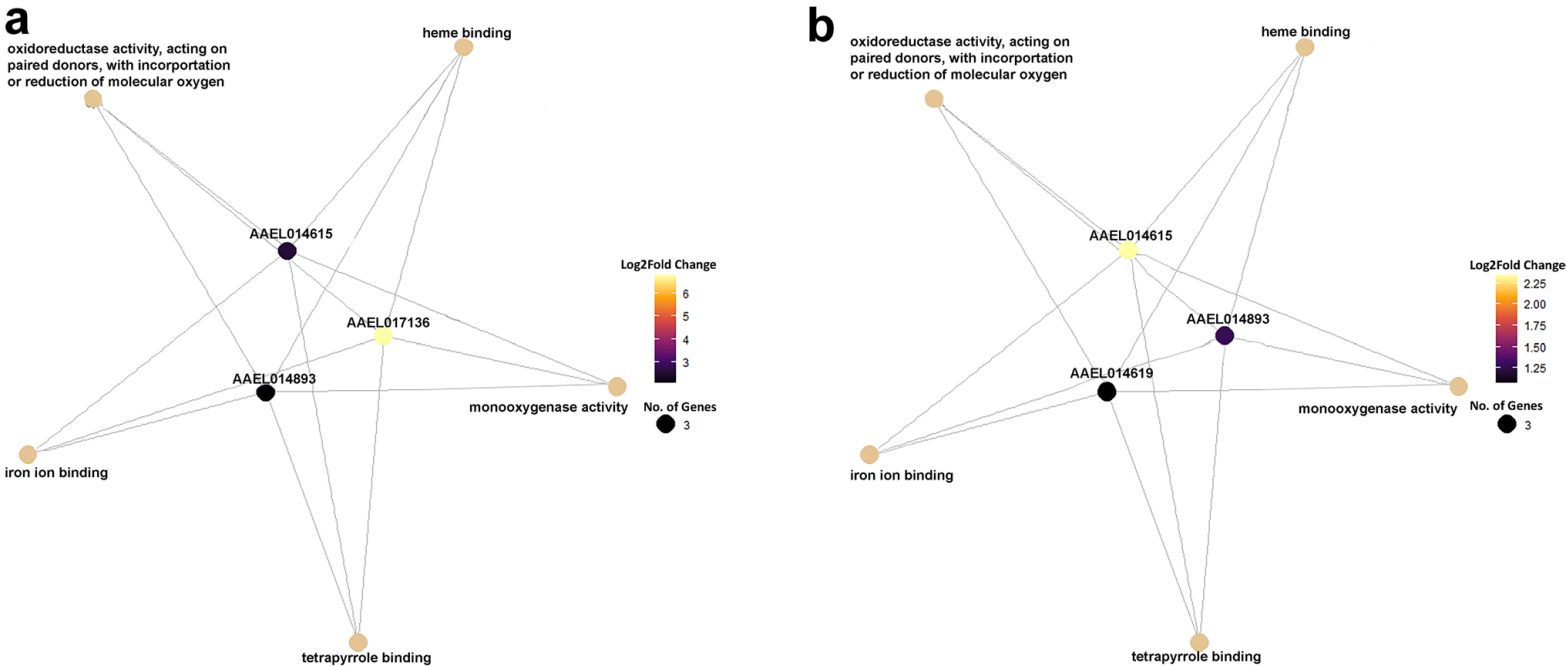
Gene concept network of over-enriched Gene Ontology (GO) molecular function (MF) terms. (A) Over-enriched GO terms associated with genes upregulated in the PR population. (B) Over-enriched GO terms associated with genes upregulated in the PRCas9 population. Functional categories are annotated based on gene annotations and/or UniProt matches (black boxes)

To identify gene networks enriched in both resistant populations, Gene Ontology (GO) enrichment analysis was performed on all differentially expressed genes (DEGs) between the resistant (PR and PRCas9) and susceptible Cas9 strains. Among the upregulated genes in the resistant strains, five molecular function (MF) GO terms were significantly enriched, while no enrichment was detected for biological process (BP) or cellular component (CC) categories. All five enriched MF terms, heme binding (GO:0020037)), monooxygenase activity (GO:0004497), oxidoreductase activity, acting on paired donors (GO:0016705), iron ion binding (GO:0005506) and tetrapyrrole binding (GO:0046906, were associated with P450 enzymes, which are well-known for their role in insecticide detoxification. Both the PR and PRCas9 population had very similar upregulated gene networks (Fig. 6A and B).

**Fig. 7:**
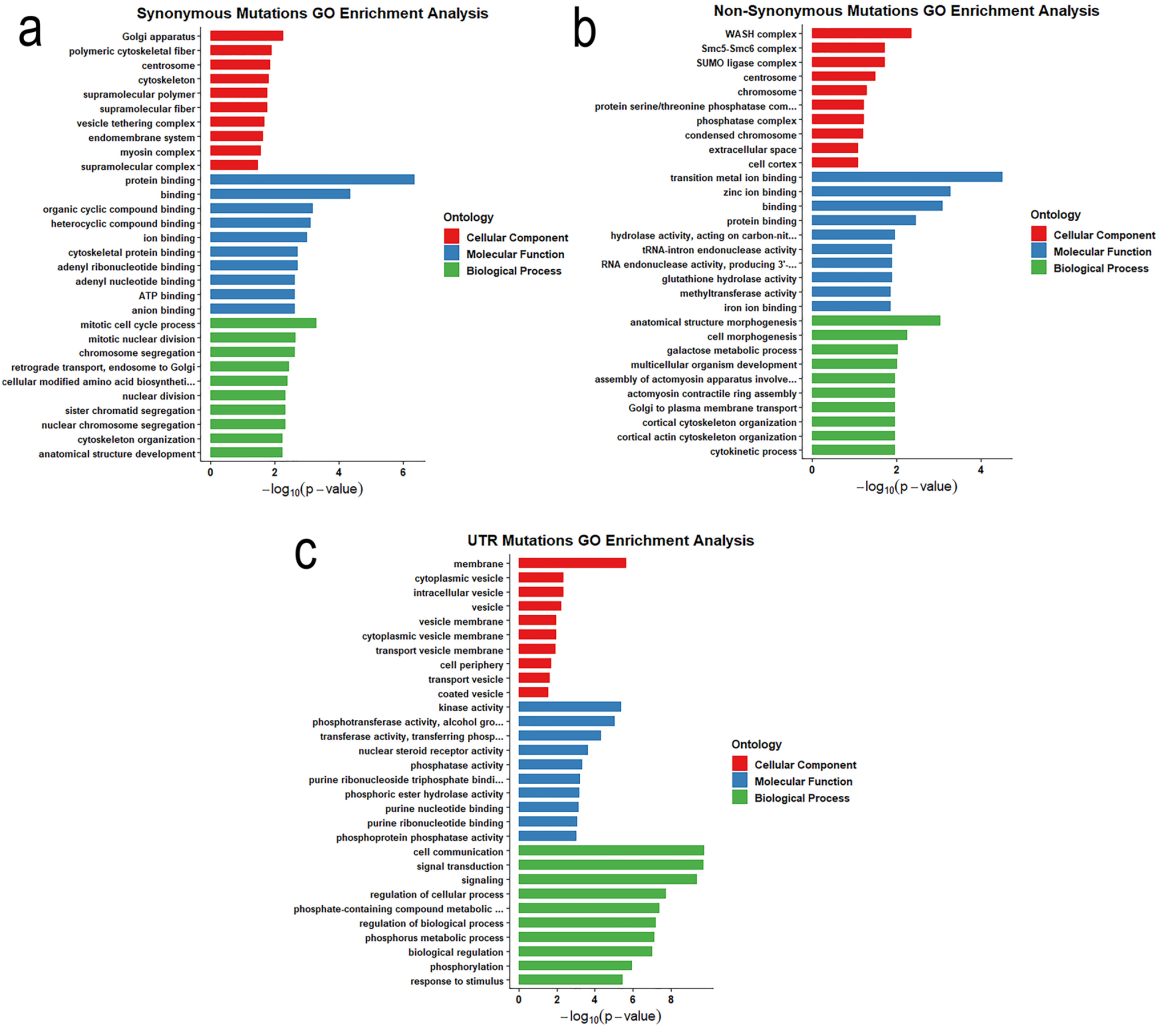
Gene concept network of over-enriched Gene Ontology (GO) molecular function (MF), biological processes (BP), and cellular component (CC) terms. Over-enriched GO terms associated with (A) synonymous, (B) nonsynonymous, and (C) 5/3 UTR variants in the PR and PRCas9 population. Functional categories are annotated based on gene annotations and/or UniProt matches (black boxes)

GO enrichment analysis was also conducted on all synonymous, nonsynonymous, and the 5’/3’ untranslated regions (UTRs) SNPs shared between the PR and PRCas9 strains, as these variants are more likely to be associated with pyrethroid resistance. Synonymous mutations were enriched in several cellular component categories related to structure organization, including polymeric cytoskeletal fiber (GO:0099513), supramolecular polymer (GO:0099081), and endomembrane system (GO:0012505) (Fig. 7a). Enriched molecular function terms among synonymous SNPs primarily involved various binding activities (GO:0005515, GO:0005488, GO:0097159, GO:1,901,363, GO:0043167, GO:0008092, GO:0032559, GO:0030554, GO:0005524, GO:0043168). The most enriched biological processes terms associated with mitosis process’ (GO:1,903,047, GO:0140014, GO:0007059, GO:0000280, GO:0000819, GO:0098813).

Nonsynonymous mutations were enriched in GO pathways distinct from those affected by synonymous SNPs. Several cellular component categories were associated with nonsynonymous variants, including the complexes of WASH (GO:0071203), Smc5-Smc6 (GO:0030915), and SUMO ligase (GO:0106068) (Fig. 7b). Nonsynonymous SNPs were also enriched in metabolic functions linked to metabolic detoxification, particularly those associated with CYP450 activities, such as transition metal ion binding (GO:0046914), iron ion binding (GO:0005506), heme binding (GO:0020037), and tetrapyrrole binding (GO:0046906). Epigenetic regulatory functions, including methyltransferase activity (GO:0008168), also contained nonsynonymous variants, suggesting possible contributions to transcriptional regulation differences between resistant and susceptible strains. In addition, biological processes related to Golgi-to-plasma membrane transport (GO:0006893) were enriched among nonsynonymous SNPs in the resistant populations.

SNPs located in untranslated regions (UTRs) showed a distinct enrichment pattern. UTR variants were overrepresented in genes associated with membrane-related components (GO:0016020, GO:0012506, GO:0030659, GO:0030658) and vesicle-associated structures (GO:0031410, GO:0097708, GO:0031982, GO:0030133, GO:0030135) (Fig. 7c). Enriched molecular functions included kinase activity (GO:0016301), transferase activity (GO:0016773, GO:0016772), and phosphatase activity (GO:0016791), indicating potential effects on signaling and post-transcriptional regulation. Within biological processes, UTR variants were found in categories related to cell communication (GO:0007154), signaling (GO:0007165, GO:0023052), and biological process regulation (GO:0050794, GO:0050789, GO:0065007).

## Discussion

Understanding the molecular basis of insecticide resistance in insect toxicology is essential for developing effective and sustainable control strategies for agriculturally, economically, and medically important insect pests. Although transcriptomic studies in *Ae. aegypti* have identified numerous DEGs, many have compared genetically distinct resistant and susceptible strains, making it difficult to distinguish true resistance mechanisms from background genetic variation (Bonizzoni et al. 2012; Reid et al. 2014; Lien et al. 2019; Derilus et al. 2023). To overcome this challenge, we generated a pyrethroid-resistant Cas9 strain (PRCas9) by repeatedly backcrossing a susceptible Cas9-expressing line with a highly pyrethroid resistant PR strain. This approach retained the strong resistance phenotype (RR > 18,000-fold) while reducing background genetic differences and enabling both transcriptomic and functional genomic analyses in a controlled genetic context. Comparison of the PR and PRCas9 strains enabled identification of resistance-associated transcriptional and variant signatures that were reproducible across resistant genetic backgrounds, strengthening inference beyond analysis of a single resistant strain.

By leveraging PRCas9 alongside the parental PR strain, we were able to isolate reproducible resistance-associated transcriptional patterns within a controlled genetic context, strengthening causal inference beyond traditional resistant–susceptible comparisons in insecticide toxicology. We also identified novel features of resistance in the PRCas9 strain, including significant upregulation of multiple CYP450s, carboxylesterases and a ROS-producing *NOX4-art*. More than 21,000 fixed SNPs unique to the resistant strains were detected, several of which occurred within detoxification genes or the promoter region of *Nox4-art*. These findings suggest that resistance in PR and PRCas9 involves both metabolic detoxification and upstream regulatory variation, potentially mediated by ROS signaling.

Transcriptomic analysis identified over 70 DEGs between resistant and susceptible populations, most of which belonged to well-established resistance pathways, particularly metabolic detoxification (P450s and esterases). The successful transfer of resistance traits to PRCas9 confirms the stability of these phenotypes across backcross generations. Reciprocal crosses produced offspring with comparable resistance levels, indicating no evidence of sex-linked inheritance—a rare but documented phenomenon in insecticide resistance (McDonald and Schmidt 1990; Zhang et al. 2019). These findings are consistent with previous work showing that the PR strain carries two homozygous *vgsc* mutations (V1016I and F1534C) and elevated P450 and esterase activities (Wang et al. 2023c). Together, these established mechanisms likely contribute to the extreme resistance phenotype retained in the PRCas9 strain and provide a biological foundation for interpreting the transcriptomic and variant data presented here.

Three P450 genes, *CYP6BB2, CYP9J19,* and *CYP9J23*, were consistently overexpressed in both PR and PRCas9 strains. These genes have been previously associated with pyrethroid resistance in *Ae. aegypti* and other species (Reid et al. 2014; Dusfour et al. 2015; Goindin et al. 2017; Ishak et al. 2017; Lien et al. 2019; Rault et al. 2019; Derilus et al. 2023; Brown et al. 2025). Among them, *CYP6BB2* has been experimentally confirmed to metabolize permethrin (Kasai et al. 2014). Although *CYP9J19* is consistently overexpressed in pyrethroid resistant mosquitoes, bacterial expression systems have not demonstrated permethrin metabolism, suggesting that insect-specific cofactors may be required (Stevenson et al. 2012). In contrast, *CYP325V1* was significantly overexpressed in the PR strain but not in PRCas9, yet both strains displayed similarly high levels of permethrin resistance. This lack of correlation suggests that CYP325V1 is unlikely to play a major role in permethrin resistance. These patterns emphasize the value of using genetically homogenized strains such as PRCas9 to more accurately identify functionally relevant resistance genes. Importantly, the development of the PRCas9 strain was intentionally designed to establish a controlled genetic framework for subsequent CRISPR-based functional validation of resistance-associated genes and regulatory elements identified here.

Carboxylesterases also contribute to pyrethroid metabolism in various insects (Feng et al. 2018). Although no single esterase gene has been definitively linked to pyrethroid resistance in *Ae. aegypti*, synergism assays (Wang et al. 2023c) suggest a moderate role for esterases in permethrin resistance. Here, *venom carboxylesterase-6* (AAEL008757) was strongly upregulated in both resistant populations (PR and PRCas9). This gene has also been implicated in pyrethroid-resistant *Anopheles stephensi* (Zhong et al. 2024), and in resistant whiteflies (Wang et al. 2023a), suggesting a potentially conserved role. Our current study detected multiple fixed nonsynonymous mutations in this gene in PR and PRCas9 (Table 5), raising the possibility that amino acid substitutions may increase its detoxification efficiency—an important target for future functional studies. Our variant analysis provides additional insight into potential mechanisms underlying resistance. Approximately half of the SNPs shared between PR and PRCas9 were synonymous and located in genes involved in membrane trafficking, binding, and protein modification. Although synonymous mutations do not alter the amino acid sequences, they can affect translation efficiency, mRNA stability, protein folding and have been linked to increasing pyrethroid resistance (Li et al. 2012; Oelschlaeger 2024). Li et al. (2012) showed positive selection of synonymous mutation variants in the *vgsc* gene of *Culex quinquefasciatus*, supporting their functional relevance. Nonsynonymous mutations, although less common, were enriched in detoxification-related genes, including metal-binding domains characteristic of P450 activity. *CYP9J23*, which was highly upregulated in both resistant populations, carried multiple synonymous and nonsynonymous SNPs, any of which may influence metabolic capacity. *CYP9J28*, a functionally validated permethrin-metabolizing P450 (Stevenson et al. 2012), also harbored nonsynonymous mutations selected in both resistant strains but never reaching fixed levels.

Variants in UTRs were enriched in genes involved in vesicle transport, cellular communication, and signaling transduction, pathways known to influence post-transcriptional regulation. UTR variants can affect mRNA stability, translation efficiency, or interactions with RNA-binding proteins (Oelschlaeger 2024). Sun et al. (2021) reported discrepancies between transcript levels and translated CYP450 proteins, underscoring a key role for UTR variants in modulating protein abundance.

Promoter variation was detected only in *CYP9J19*; the other upregulated CYP450s showed no detected promoter changes. This pattern suggests that their increased expression is more likely driven by *trans-*regulatory mechanisms rather than by *cis*-regulatory variation within their own promoter regions. However, because variants were derived from transcriptomic alignments, structural variation such as copy number variation (CNV), gene duplication, or paralog cross-mapping may also contribute to both increased expression and apparent SNP patterns. CNV and gene duplication have been widely reported as important mechanisms underlying metabolic insecticide resistance in mosquitoes, particularly for detoxification gene families such as cytochrome P450s and esterases (Faucon et al. 2015; Lucas et al. 2019). In contrast, the promoter of *NOX4-art* contained 8 SNPs and a six-base-pair deletion unique to resistant populations. *NOX4-art* encodes an NADPH oxidase that produce H₂O₂, a reactive oxygen species involved in ROS signaling pathways (Gandara et al. 2017). Increased *NOX4-art* expression in PR and PRCas9 may elevate ROS levels and contribute to the transcriptional activation of detoxification genes through ROS-mediated regulatory cascades (Li et al. 2024; Zhou et al. 2025). Although descriptive, these findings provide mechanistic hypotheses for future testing using CRISPR-based functional assays in the PRCas9 genetic background.

Given the pronounced overexpression of detoxification genes in toxicological responses, particularly P450s, understanding their upstream regulation is essential. Multiple studies have showed ROS–CncC/Maf, MAPK–CREM, and G-protein/cAMP/PKA pathways can activate detoxification gene expression across insect taxa (Goldsmith and Dhanasekaran 2007; Yang et al. 2020; Liu et al. 2021; Lu et al. 2021; Fu et al. 2024; Li et al. 2024; Tang et al. 2020). The marked upregulation and promoter variation of *Nox4-art* observed here aligns with these findings and suggests a potential upstream regulatory role that warrants further investigation.

## Conclusion

This study clarifies the molecular mechanisms underlying permethrin resistance in *Ae. aegypti* in insect toxicology by combining transcriptomic profiling with variant analysis in a genetically similar resistant background. The PRCas9 strain, developed through repeated backcrossing with the highly resistant PR population, retained extreme resistance while allowing direct comparison to a susceptible Cas9 line. Across both resistant strains, we identified consistent overexpression of key detoxification genes—particularly P450s and carboxylesterases—and detected numerous fixed SNPs, including promoter variants unique to resistant populations. Notably, elevated expression and promoter variation of *NOX4-art* suggest a potential role for ROS-mediated regulatory pathways in driving detoxification gene activation. Together, these findings provide a refined list of candidate genes and regulatory elements involved in pyrethroid resistance and establish PRCas9 as a powerful genetic platform for future CRISPR-based functional validation and the development of targeted vector control strategies.

## Supplementary Information

Below is the link to the electronic supplementary material.

Supplementary file1 (PDF 137 kb)

## Data Availability

The RNA-seq data supporting the findings of this study have been deposited in the NCBI Sequence Read Archive (SRA) under BioProject accession PRJNA1282039. Additional data supporting the conclusions of this study are available from the corresponding author (NL) upon reasonable request.
