# Supplementary material for: Integrated transcriptomic and variant analysis reveals molecular mechanisms of pyrethroid resistance in a genetically homogenized Cas9 strain of Aedes aegypti
Source: Arch Toxicol. 2026 Mar 16;100(6):2543–58. doi: 10.1007/s00204-026-04326-x (PMC13221410; doi:10.1007/s00204-026-04326-x)
Supplement: Supplementary file 1 — Supplementary file1 (PDF 137 kb) [file 204_2026_4326_MOESM1_ESM.pdf]

Archives of Toxicology

Integrated Transcriptomic and Variant Analysis Reveals Molecular Mechanisms of Pyrethroid Resistance in a Genetically Homogenized Cas9 Strain of *Aedes aegypti*

Dylan Brown, Reese Houck, and Nannan Liu*

Department of Entomology and Plant Pathology, School of Agriculture, Auburn University, Auburn, AL, 36849.

*Corresponding author

Email Address of Authors:

Dylan Brown, djb0094@auburn.edu

Reese Houck, rah0106@auburn.edu

Corresponding author:

Nannan Liu, liunann@auburn.edu

Supplementary Table S1. Primers used in this study

Primer Name	Sequence (5’-3’)
CYP325v1 Promoter Fwd	CACTGATCCTTCCGGTTGTCC
CYP325v1 Promoter Rev	GGAAAAGCTTCTCTCATACTTGAC
CYPJ23 Promoter Fwd	CGTGGCCGTGCGGTTAGC
CYPJ23 Promoter Rev	GACCACGATTGCTGCGATC
CYP9J22 Promoter Fwd	GGAAGGCACCAAACTGATCG
CYP9J22 Promoter Rev	CGCCAATACGGCTACTACG
CYP9J19 Promoter Fwd	GGAACAACGAGAAGCACACAAG
CYP9J19 Promoter Rev	GAATAACTATGCAGCCAACGG
NOX4-Art Promoter Fwd	CGCAGACTTCATCCTTTGGCGG
NOX4-Art Promoter Fwd	CCAAATCGAGGCTAAGCGGGTC
CYP6BB2 Promoter Fwd	CGGCGAGAGCTCAATGTATC
CYP6BB2 Promoter Rev	GAAGTAGACGAGCCCCGTTAG
CYP325v1 qRT-PCR Fwd	TTTACCGGTTGACCAGCGAT

CYP325v1 qRT-PCR Rev	ACATTCGGGGTCGCTGTTGT
CYP9J23 qRT-PCR Fwd	GTGCACCTTTCCGGAGTTTA
CYP9J23 qRT-PCR Rev	AAGGGTCTTCTCGAACAGCA
CYP6BB2 qRT-PCR Fwd	TAGTCGCTAAGGACGGAGGA
CYP6BB2 qRT-PCR Rev	AAGTACTCCGGATCGTGGTG
CYP9J19 qRT-PCR Fwd	GACCAAACCGGTGTTCATGC
CYP9J19 qRT-PCR Rev	TAGCTGATGGAAGGGACCGA
CYP9J22 qRT-PCR Fwd	TGACAGCGTGTCATCCGAAA
CYP9J22 qRT-PCR Rev	GCGTATCGTATGTCGGTGGT
Venom qRT-PCR Fwd	TTGCTAGGACGGGAGATCCA
Venom qRT-PCR Rev	TTAGTTGACTGCCGGAAGCG
AAEL023346 qRT-PCR Fwd	CTAGTCCATAAAACCCGCTGC
AAEL023346 qRT-PCR Rev	CCTGTACCTGTCCTCCAGTTG
NOX4-art qRT-PCR Fwd	GGACAGGCGAAAAGTATCCA
NOX4-art qRT-PCR Rev	GACTGTAAACGGGTGCCACT
AAEL003195 qRT-PCR Fwd	CTAGTCCATAAAACCCGCTGC
AAEL003195 qRT-PCR Rev	CCTGTACCTGTCCTCCAGTTG
Venom SNP Fwd1	GATATGCCAACGCACCAGTAG
Venom SNP Rev1	CCAGTACTCATGAAACCGAGC
Venom SNP Fwd2	CCATCGAGTTATTGTGATGAGTG
Venom SNP Rev2	GGTGGTCCCAGAAAGAGAATC
CYP9J23 SNP Fwd1	GATTCCTACCACGTCAACAAGTGTG
CYP9J23 SNP Rev1	CAACACTCAGCCTAGTCAGCCG
CYP9J28 SNP Fwd	GGACCAAAGGTGTACGAGATG
CYP9J28 SNP Rev	CGTGTCGAACCCTGCTAGG
Carboxylic ester hydrolase SNP Fwd1	GGGTTGTTGGTTTACTCGCAG
Carboxylic ester hydrolase SNP Rev1	CCAGCTCCTGCAGACTGTC
Carboxylic ester hydrolase SNP Fwd2	CCTCGAAAATGATCAACGACAC
Carboxylic ester hydrolase SNP Rev2	CCACTACGGTCACGAATAACAC
ABC Transporter SNP Fwd	GGCATCAAAAGCCTGCACTG
ABC Transporter SNP Rev	GCAACACGGTCAGAGTGAAG
